# Editorial: Highlights in diagnostic and therapeutic devices 2021/22

**DOI:** 10.3389/fmedt.2023.1119558

**Published:** 2023-02-22

**Authors:** Yangzhi Zhu, Mohammod Abdul Motin, Qingyu Cui

**Affiliations:** ^1^ Terasaki Institute for Biomedical Innovation, Los Angeles, CA, United States; ^2^ Department of Electrical and Electronic Engineering, Rajshahi University of Engineering & Technology, Rajshahi, Bangladesh; ^3^ Department of Medicine, David Geffen School of Medicine, University of California-Los Angeles, Los Angeles, CA, United States

**Keywords:** diagnostic, therapeutic, flexible electronics, biosensors, personalized healthcare

**Editorial on the Research Topic** Highlights in diagnostic and therapeutic devices 2021/22

## Introduction

Developing flexible systems that monitor human health and deliver feedback therapy are the unmet medical needs for applications of personalized medicine and digital healthcare (1, 2). Several representative examples include wearable/implantable electronics (3), (bio)sensors (4), functional materials (5), advanced algorithms (6), and manufacturing (7). Such technological development and advancements have accelerated the significant breakthrough in multiple biomedical studies, such as early-stage disease diagnostics/prevention, intelligent drug-delivery systems, novel theranostic devices, and clinical imaging (8–10).

We are pleased to see the quality of the research submitted to our topic in diagnostic and therapeutic devices. This Research Topic finally contains six research and two review articles that address the recent advances and insights in diagnostic and therapeutic devices from scholars in a variety of countries (*i.e.*, United States, United Kingdom, Germany, Canada, France, Italy, Spain, Israel, Turkey, and Thailand) and nominated universities/institutions (*i.e.*, Massachusetts Institutes of Technology (MIT), University of California, Los Angeles (UCLA), University of Pennsylvania (UP), Tufts University, and Weill-Cornell Medicine,). Herein, we provide a brief introduction of these published articles and welcome readers to refer to these publications and their associated references for more details on this Research Topic.

Vėbraitė et al. summarized the recent advancements in the field of high-resolution neurostimulation from a technological perspective and the future opportunities stemming from materials science and engineering to make smaller, softer, and higher electrode density devices for neural stimulation and recording. Kenny et al. illustrated the physiological rationale and provided proof-of-concept data to suggest that simultaneous venous and arterial Doppler should be used to infer the slope of the Frank-Starling curve. Such a hypothesis is based on the phenomenon that the Frank-Starling slope changes rapidly in the ICU as a function of disease and therapy. Letellier et al., report in their pilot study that it is possible to automatically detect asynchrony events (AEs) from sole pressure and flow waveforms by using SYNCSMART software and that the results are within the inter-rater variability, providing a reliable assessment of patient-ventilator asynchrony. Afra et al. offer a comprehensive evaluation of the Vagus nerve stimulation (VNS) therapy system technology for drug-resistant epilepsy, including the technological evolution of the VNS with device approval milestones and a comparison of conventional open-loop and closed-loop generator models. This review could potentially serve as a valuable resource as this anti-epileptic neuromodulation technology continues to evolve. Basnet et al. report examining the field of energy-based medical therapies based on the analysis of patents, indicating an increasingly important role for energy-based therapies in the future of medicine. Prezelski et al. propose a novel multi-point injection cannula for achieving broader volume distribution than current single-needle designs and validate this system in benchtop studies of trypan blue convention enhance delivery in agarose brain phantoms, demonstrating a significant increase in volume distribution compared to single-needle delivery, while significantly reducing the total duration of the procedure. Schumayer et al. present an interesting three-dimensional masking process and demonstrate its successful application for the partial coating of a scleral contact lens electrode. Such techniques could open up more applications, including the mass production of brain electrodes or the structuring of microfluidic systems. Sringean et al. demonstrate the technical feasibility of using multisite wearable sensors to quantitatively assess early objective outcome measures of the ability of patients with Parkinson's disease to get out of bed, which significantly correlates with axial severity scores, indicating that axial impairment could be a contributing factor in difficulty getting out of bed.

The articles published on this Research Topic cover a broad spectrum of research in wearable/implantable diagnostic and therapeutic devices that will bring new solutions and perspectives for the next-generation medical devices. We sincerely thank all the authors for their contributions to this Research Topic. It is also essential to acknowledge all the reviewers' time and assistance in ensuring the scientific validity and quality of the work submitted to this Research Topic.
